# The measured healthy lifestyle habits among Saudi university females in Medina, Saudi Arabia: A cross-sectional study

**DOI:** 10.1097/MD.0000000000038712

**Published:** 2024-07-05

**Authors:** Amal M. Qasem Surrati, Eilaf Hasan Altayeb, Wedyan Ali Almohammadi, Rahaf Mustafa Aljohani, Haya Shaher Ali Altouri, Rahaf Abdullah Alhawsawi

**Affiliations:** a Family and Community Medicine Department, College of Medicine, Taibah University, Medina, Saudi Arabia; b College of Medicine, Taibah University, Medina, Saudi Arabia.

**Keywords:** International Physical Activity Questionnaire, lifestyle habit, Pittsburgh Sleep Quality Index, Saudi university female, unhealthy diet, well-being index

## Abstract

Lifestyle plays a crucial role in shaping an individual’s health outcomes, we aim to calculate the prevalence of lifestyle habits among female populations in the College of Medicine, Taibah University including poor dietary habits, lack of physical activity (PA), poor coping with stress, and impaired sleep patterns and to find factors that are correlated to them. A cross-sectional study was conducted among Saudi females at the College of Medicine, Taibah University, from January 1 to June 1, 2023. Data were collected through interviewing them using validated questionnaires assessing 5 different lifestyle domains. Statistical analysis was performed using Statistical Package for the Social Sciences version 21.0. A total of 263 cases were interviewed. The mean age was 22 ± 8.4 years old. The average sleep quality measured by the Pittsburgh Sleep Quality Index of 2.6 ± 1.1, suggesting relative difficulty in sleep quality. A total of 68.6% participated in moderate PA. Dietary habits indicated a high prevalence of consumption of sweets, and fast meals, alongside low intake of fruits and vegetables. Emotional well-being, as assessed by the World Health Organization-5 questionnaire, yielded an average score of 7.8 ± 5.7, 58.9% moderate stress, and 8% high perceived stress. Adequate sleep quality is crucial for well-being, necessitating lifestyle modifications, particularly weight management, to address sleep disorders. Varied PA levels (46% meeting recommendations) highlight the need for standardized guidelines and tailored interventions. The high prevalence of unhealthy dietary habits underscores the importance of targeted nutritional interventions. Stress prevalence (40%) emphasizes the need for individualized stress management strategies.

## 1. Introduction

The person’s way of life is shaped by the characteristics of the person’s actions within the area he lives which include his regular functions in his job, hobbies, leisure activities, and diet.^[1]^ These actions affect 60% of his health as they are linked to lifestyle choices.^[2]^ Following an unhealthy lifestyle may lead to challenging conditions like disability, anxiety, fatigue, and other related challenges.^[3]^ Also, it increases the risk of developing comorbidities like obesity, heart conditions, vascular problems, joint ailments, skin roughness, mood disorders, and more.^[4]^

Lifestyle medicine involves employing evidence-supported behavioral strategies to prevent, treat, and handle chronic illnesses which in turn improve overall health and well-being.^[5]^ These factors include a healthy diet primarily focused on whole, plant-based foods, regular physical activity (PA), quality sleep, stress control, refraining from harmful substances, and fostering positive social relationships – which also serves as a powerful preventive measure against these health conditions.^[6]^

Sleep disturbances significantly impact the overall quality of life. Subpar sleep quality is widespread among the Saudi population as it affects 41% with a prevalent trend of insufficient sleep duration among 1 in every 3 Saudi adults.^[7]^ However, extended sleep periods exceeding 9 hours were found to correlate with increased comorbidities risk.^[8]^

The uncontrolled diet observed in the Saudi population provides insights about the risks of developing obesity or diabetes mellitus as about 28% of them are overweight. In comparison, 20% are obese^[8]^ which can be explained by poor dietary practices like regular snacking and insufficient fruit and vegetable intake, which are common habits among the Saudi public.^[5]^ Also, other bad habits increase the risk of obesity like skipping breakfast, frequently eating fast food, having a low daily intake of milk and dairy products, and regularly consuming sweets or carbonated drinks.^[9,10]^

Stress also affects health outcomes by increasing the risk of cardiac diseases, obesity, and type 2 diabetes^[11]^ which can be explained by the secretion of hormones, peptides, and neurotransmitters that are involved in the body’s response to stress especially if the stress is severe and prolonged.^[12]^ Also, physical inactivity has a role in deteriorating health outcomes, increases the risk of obesity,^[13]^ and poses a significant challenge in Saudi Arabia as its prevalence ranges from 43.3% to 99.5%, among the Saudi population.^[14]^

All these lifestyle factors are connected and affect well-being as exercise, a healthy diet, and good sleep are pivotal elements in improving health and managing stress.^[15]^ Exercise, besides its multifaceted benefits in enhancing physical fitness and cardiovascular health, also improves mental well-being by reducing stress, anxiety, and depression as it promotes the release of endorphins, contributing to an improved sense of well-being.^[16]^ Parallelly, dietary patterns affect overall health as a diet comprising fruits, vegetables, whole grains, and lean proteins not only supports physiological functions but also aids in mood regulation and stress management. Also, sleep is related to overall well-being as consistent and restorative patterns are associated with improved cognitive function, emotional resilience, and a bolstered immune system.^[17]^

Assessing well-being through indices is a valuable tool in evaluating an individual’s holistic health. These indices encapsulate various aspects of life, including physical health, mental state, social connections, and environmental factors, providing a comprehensive picture of an individual’s overall wellness. Stress, a ubiquitous element in modern life, profoundly affects an individual’s well-being. Chronic stress negatively affects both physical and mental health. Understanding stressors and implementing strategies to mitigate stress levels are paramount in fostering a balanced and healthy lifestyle.^[18]^

In this study, we aim to calculate the prevalence of lifestyle habits among female populations in the College of Medicine, Taibah University including poor dietary habits, lack of PA, poor coping with stress, and impaired sleep patterns, and to find factors that are correlated to them.

## 2. Methodology

### 2.1. Study design

We conducted a descriptive cross-sectional study among Saudi females in the College of Medicine, Taibah University from January 1, 2023, to June 1, 2023. The inclusion criteria were female medical students in Phase 1 including (2nd and 3rd years), phase 2 including (4th and 5th year), and Phase 3 including 6th year female college academic staff; and female employees while the exclusion criteria were those who refused to participate, and pregnant ladies.

### 2.2. Ethical considerations

We conducted our study after receiving ethical approval from the Taibah University Medical College Research and Ethics Committee by this ID: IRB00010413. Also, all participants signed informed consents electronically before joining our study, they were aware of the objective of the study, and the confidentiality of their responses was assured.

### 2.3. Sample size assumptions

According to the registry of female colleges, the total number of students was 747 and the summation of academic staff and administrative employees was 82, therefore, the total population present in 2023 was 829.

The sample size was determined by using the SampSize software online for prevalence studies of a total population of 829 and at a 95% confidence level. The following inputs yielded a sample size of at least 263 participants: precision = 5.00 %, prevalence = 50.00 %, population size = 829, and 95% confidence interval specified limits (45% [these limits equal prevalence plus or minus precision]).

Then, the sample was collected using a non-probability convenience sampling technique by including the individuals who were accessible to the researcher and agreed to participate. This resulted in 206 participants from students at different academic years and 54 participants from employees. At first, we conducted a pilot study by interviewing 10 to 15 students to test the applicability of our study.

### 2.4. Data collection methods

The interviews were conducted after academic classes and duties during the 6 months started in January to June 2023 using the questionnaires attached which consist of 3 parts: the first part consisted of demographic questions like age, nationality, marital status, residency, occupational status, and monthly income; the second part consisted of diet habits by assessing the quality of diet, exercise assessed by the 7 items of International Physical Activity Questionnaire (IPAQ), stress assessed by 10 items of Perceived Stress Scale (PSS), emotional well-being assessed by 5 items of World Health Organization (WHO) Well-Being Index questionnaire, sleeping patterns assessed by 9 items of the Pittsburgh Sleep Quality Index (PSQI), and smoking by assessing the tobacco pattern use; and the third part consisted of the clinical examination including weight, height, waist circumference (WC), and blood pressure.

We collected the clinical data as follows: We used a validated portable blood measurement device and the final measurement was taken by the average of 3 measurements taken 5 minutes apart during rest. Normal systolic blood pressure was defined as <130 mmHg. Regarding weight, the individuals weighed in bare feet and light indoor clothing using an electronic scale device and it was recorded to the nearest 0.1 kg. Regarding height, it was measured without shoes using a portable studio meter and recorded to the nearest 0.5 cm. WC: was assessed 2 times to the nearest 0.5 cm, with a flexible but non-elastic measuring tape, and was determined at the level of the natural waist (the tightest part of the torso) or 1 finger width under the umbilicus. A normal WC was ≤35 inches for women. Body mass index (BMI) was calculated based on height and weight using this formula: weight in kg ÷ height in m^2^.

### 2.5. Data management and analysis plan

Data were coded, entered, and analyzed using the Statistical Package for Social Science version 21.0 (SPSS, Chicago). Quantitative data were represented by mean and standard deviation and qualitative data were represented by frequencies and percentages. Descriptive statistics were used to determine the prevalence of mental illness. The significance of associations was tested using Chi-square for categorical variables and Student *t* test for continuous variables. The test results were considered significant when *P* value < .05.

## 3. Results

### 3.1. Baseline characteristics of patients

A total of 263 subjects (Employed 54 [20.5%] and Students 209 [79.5%]) were interviewed. Table 1 shows a description of the sample. Most individuals were aged <59 years (mean age 22 ± 8.4 years old). Most of the participants were singles (85.9%), were satisfied with their income (76.8%), were overweight (50.6%), had a mean WC of 88 ± 16, had a mean waist/hip ratio of 0.86 ± 0.7, were nonsmokers (95.8%) Supplementary Material S6, Supplemental Digital Content, http://links.lww.com/MD/N79, were not on medical treatments (92.4%), had anxiety (18.6%), or were relatively stressed (54.4%).

**Table 1:** Socio-demographic characteristics of Saudi adult women.

Variables	Women (n = 263)
Age in yr (mean ± SD)	22 ± 8.4
Marital status	
Married	30 (11.4%)
Widow	3 (1.1%)
Divorced	4 (1.5%)
Single	226 (85.9%)
Employment status	
Employed	54 (20.5%)
Students	209 (79.5%)
Educational level	
Less than high school	6 (2.3%)
High school	157 (59.7%)
University	94 (35.7%)
Postgraduate studies	6 (2.3%)
Medical students’ study level (n = 209)	
Phase 1	100 (47.8%)
Phase 2	54 (25.9%)
Phase 3	55 (26.3%)
Income	
Unsatisfied	16 (6.1%)
Satisfied	202 (76.8%)
Satisfied and more	45 (17.1%)
How do you describe your lifestyle?	
Very stressful	32 (12.2%)
Stressful	74 (28.1%)
Relatively stressful	**143 (54.4%**)
Free from stress	14 (5.3%)
Presence of chronic disease	
No chronic disease	122 (46.4%)
Diabetes mellitus	12 (4.6%)
Hypertension	13 (4.9%)
Coronary heart disease	5 (1.9%)
Dyslipidemia	6 (2.3%)
Chronic renal disease or/failure	2 (0.8%)
Inflammatory disease (rheumatoid arthritis).	7 (2.7%)
Depression	31 (11.8%)
Anxiety disorder (diagnosed)	**49 (18.6%**)
*Others	16 (6.1%)
BMI (kg/m^2^)	
Underweight (<18.5)	37 (14.1%)
Normal weight (18.5–25)	53 (20.2%)
Overweight (25–29.9)	**133 (50.6%**)
Obese (≥30)	39 (15.2%)
Waist circumference WC (mean ± SD)	**88 ± 16**
WC (≤88 cm)	120 (45.6%)
WC (>88 cm)	**143 (54.3%**)
Blood pressure	125/80 ± 16.1
Tobacco smoking	Nonsmokers = 252 (95.8%) Former smoking = 5 (1.9%) Current smoking = 6 (2.3%)
Waist/hip ratio (mean ± SD)	**0.86 ± 0.7**
≤0.85	119 (45.2%)
>0.85	**144 (54.8%**)
Medical treatments and supplements	
†Yes	20 (7.6%)
No	243 (92.4%)

Numbers (%) are shown. Mean ± SD (SD). The bold indicates the highest percentages or mean.

BMI = body mass index, WC = waist circumference.

* Others included having >1 health problem and those with other diseases such as thyroid disorders, osteoporosis, gastrointestinal disorders, anemia, etc. †Medical treatments and supplements (YES) such as Aspirin and statin drugs.

### 3.2. PSQI Questionnaire related to quality of sleep

The PSQI comprises a set of 19 items that individuals self-rate (Supplementary Material S1, Supplemental Digital Content, http://links.lww.com/MD/N74) The scoring process only takes into account questions that are self-rated. The 19 elements that participants assessed themselves on are aggregated to create 7 distinct scores, referred to as “components,” with a scoring range of 0 to 3 points. Our results indicated that 44.5% needed 30 to 60 minutes to fall asleep and 51.7% described their sleep quality as fairly bad (Table 2).

**Table 2:** Descriptive statistics for components, global PSQI (n = 263).

Variables	Mean	SD	Range
1.Subjective sleep quality	**2.6 relatively difficulty**	1.1	0–3
2.Sleep latency	1.7	0.9	0–3
3.Sleep duration	**2.5 relatively difficulty**	1.1	0–3
4.Sleep efficiency	**3 significant difficulties**	1.2	0–3
5.Sleep disturbances	1.4	0.9	0–3
6.Day-time dysfunction	1.2	0.7	0–3
7.Sedative usage	1.1	0.1	0–3
A global score	**6.00**	2.1	1–19
*Sleep efficiency (%).	**45.2%**	10.2	0–100

Mean ± SD are shown.

PSQI = Pittsburgh Sleep Quality Index.

* Calculate the habitual sleep efficiency with the following equation: (Hours slept [#1]/h spent in bed [result above])*100 = habitual sleep efficiency (in %).

Seven components are equally weighted on the PSQI questionnaire’s 19 items: subjective sleep quality (1 item), sleep latency (2 items), sleep duration (1 item), sleep efficiency (3 items), sleep disturbances (9 items), daytime dysfunction (2 items), and sedative use (1 item). In every instance, a score of “0” signifies the absence of difficulty, whereas a score of “3” signifies the presence of significant difficulty. In our subjects, the data indicated poor overall sleep duration and efficiency among participants with relative difficulty (2.6 ± 1.1) according to sleep quality.

The summation of the 7 component scores results in a single “global” score, which spans from 0 to 19 points. A score of 0 signifies the absence of any issues, while a score of 19 indicates serious difficulties across all categories (Main 1). Also, the global PSQI score was derived by summing the values of the 7 components, which comprised the scale’s conceivable range of scores from 0 to 19. A global score >5 signifies an inadequate SQ (6 ± 2.1) which was determined in our subjects with 45.2% sleep efficiency, sleep efficiency of <80% was thought to be reduced sleep efficiency (normal sleep efficiency is considered to be 80% or greater, (Table 2).^[19]^

### 3.3. IPAQ Questionnaire related to the quality of exercise

The PA categories of the sample are presented in (Supplementary Material S2, Supplemental Digital Content, http://links.lww.com/MD/N75), according to the evaluating procedure of the IPAQ. In all, 91 individuals, accounting for 34.6% of the sample, engaged in vigorous physical activities, whereas the other 172 participants, constituting 65.4% of the total sample size of 263, did not partake in such activities. Among the sample of 172 participants, a majority of 118 individuals (68.6%) engaged in moderate PA. Among the total sample size of 263 participants, a significant majority of 205 individuals (77.9%) engaged in walking activities for a minimum duration of 10 minutes. In aggregate, the subjects achieved a total of 1200 MET of task minutes each week as a moderate-intensity activities indicator (Supplementary Material S2, Supplemental Digital Content, http://links.lww.com/MD/N75).

According to Hallal et al, a total PA score was computed as

Walking MET-min/wk = 3.3 * walking minutes * walking days

Moderate MET-min/wk = 4.0 * moderate-intensity activity minutes * moderate days

Vigorous MET-min/wk = 8.0 * vigorous-intensity activity minutes * vigorous-intensity days

Total PA MET-min/wk = sum of walking + moderate + vigorous MET

min/wk scores.^[20]^

**Figure 1.**
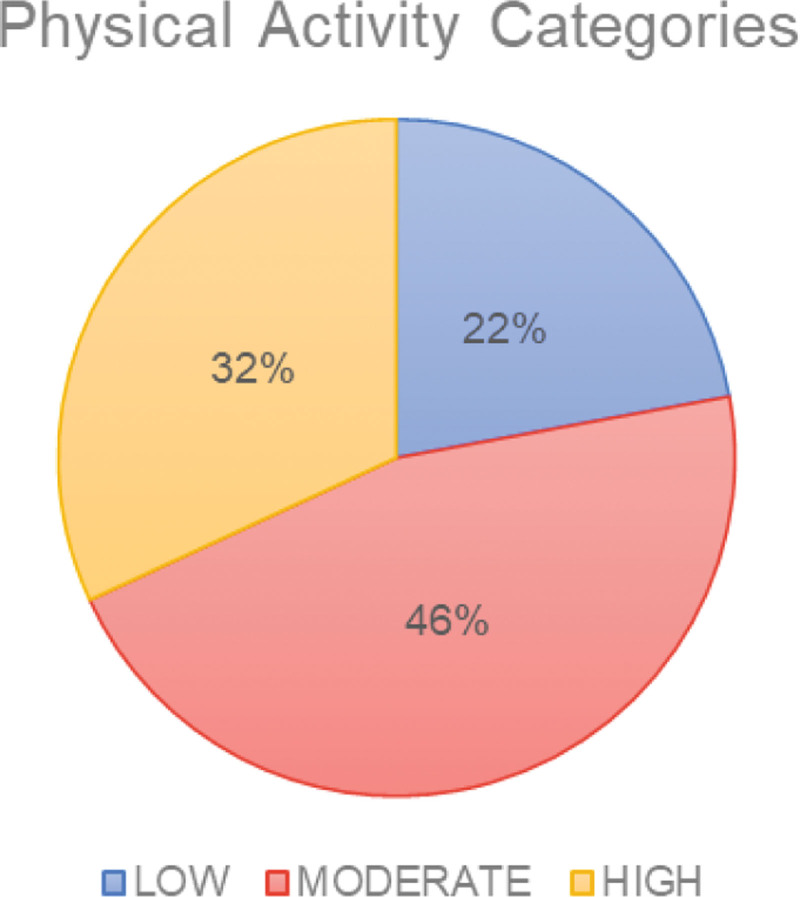
Physical activity categories.

In general, 46% of the participants achieved the recommended level of at least 500 minutes per week of moderate PA for 5 days, which is considered a reasonable threshold for obtaining health benefits (Supplementary Material S2, Supplemental Digital Content, http://links.lww.com/MD/N75, Fig. 1, Table 3).

**Table 3:** IPAQ calculations (n = 205 who did activities vigorous, moderate, and walking).

High activity criteria		Moderate activity criteria			
≥3 d vigorous (≥1500 min/wk) 44 (21.5%)	≥7 d any (≥3000 min/wk) 40 (19.5%)	≥3 d vigorous (≥20 min/d) 39 (19%)		≥5 d mod/walk (≥30 min/d) 51 (24.9%)	≥5 d any (≥600 min/wk) 31 (15.1%)
Physical activity categories (n = 263)					
Low			58 (22.1%)		
Moderate			**121 (46%**)		
High			84 (31.9%)		

Numbers (%) are shown.

Low: If not moderate or vigorous.

Moderate: (a) 3 or more days of vigorous-intensity activity of at least 20 minutes per day, (b) 5 or more days of moderate-intensity activity and/or walking of at least 30 minutes per day, (c) 5 or more days of any combination of walking, moderate-intensity or vigorous-intensity activities achieving a minimum T = total physical activity of at least 600 min/wk.

High: (a) Vigorous-intensity activity on at least 3 days (20 min minimum, achieving a minimum Total physical activity of at least 1500 min/wk, (b) 7 or more days of any combination of walking, moderate-intensity or vigorous-intensity activities achieving a minimum Total physical activity of at least 3000 min/wk.

IPAQ = International Physical Activity Questionnaires.

Participants were classified as low PA for 58 participants (22.1%), moderate PA for 121 participants (46%), and high PA for 84 study subjects (31.9%).

### 3.4. Questionnaire related to quality of diet

As in Supplementary Material S3, Supplemental Digital Content, http://links.lww.com/MD/N76 presents data on the high prevalence of frequent consumption of sweets, fast meals, and snacks, as well as low portions of fruits and vegetables per day among the participants. Additionally, it highlights the high percentage of participants who reported regular intake of sugar and honey in their tea, coffee, or other beverages.

- 41.4 % eat sweets such as Halwa, Kunafa, and cookies 6 times per week.
- 38.4% eat fried foods such as French fries and chicken strips 5 times per week.
- 39.5% eat high-salt snacks such as chips and nuts 5 times per week.
- 29.7% consume sugar and honey in tea, and coffee 5 times per week.
- 31.2% eat refined food items like burgers, and pizza 4 times per week.
- 39.5% eat fruit and salad 1 times per week.
- 31.2% eat sprouted pulses and green vegetables 1 times per week.

### 3.5. WHO (Five) Well-Being Index questionnaire related to the quality of emotional well-being

As in Supplementary Material S4, Supplemental Digital Content, http://links.lww.com/MD/N77, the response data from the Well-Being Index Questionnaire indicates that almost a third of our sample experienced <50% of their time feeling pleasant, peaceful, active, and waking up feeling refreshed, 31.6% felt cheerful and in good spirits, 31.9% felt calm and relaxed, 31.6% felt active and vigorous, 25.9% woke up feeling fresh and rested, and 27.8% felt that their daily life has been filled with interesting things.

WHO-5 Well-being Index is a frequently used instrument for depression screening that consists of 5 items scored on a 6-point Likert scale as follows: at no time (0), some of the time (1), less than half of the time (2), more than half of the time (3), most of the time (4), and all of the time (5). The scale runs from 0 to 25 (worst well-being to highest well-being). The lowest response for each question was 1 and the highest response was 5. Each question had a response scale ranging from 1 to 5 (Table 4). The average score of the WHO-5 questionnaire was 7.8, with an SD of 5.7. The item with the lowest mean score was item 4, with a mean score of 1.4 and an SD of 1.1. Conversely, item 1 had the highest mean score of 2.3, with an SD of 1.2 (Table 4).

**Table 4:** Well-Being Index Questionnaire’s scoring (n = 263.)

WHO-5	Mean	SD
1. I have felt cheerful and in good spirits	2.3	1.2
2. I have felt calm and relaxed	1.1	1.1
3. I have felt active and vigorous	2	1.1
4. I woke up feeling fresh and rested	1.2	1.1
5. My daily life has been filled with things that interest me	1.5	1.2
Total score	**7.8**	**5.7**

WHO-5 = World Health Organization-5 Well-Being Index.

### 3.6. PSS-10 questionnaire related to the quality of stress

As in Supplementary Material S5, Supplemental Digital Content, http://links.lww.com/MD/N78 displays the item analysis conducted on the responses of the research participants for the PSS. A significant proportion of our study participants, above 40%, reported experiencing sometimes of felt stress in their lives.

- In the last month, 40.7% have been upset because of something that happened unexpectedly.
- In the last month, 42.6% felt they were unable to control the important things in their life.
- In the last month, 41.8% often have felt nervous and stressed.
- In the last month, 38% often have felt confident about their ability to handle their problems.
- In the last month, 37.6% often felt that things were going their way.
- In the last month, 46.4% often have found their selves could not cope with all the things that they had to do.
- In the last month, 42.2% often have been able to control irritations in their life.
- In the last month, 38% often have felt that they were on top of things.
- In the last month, 42.2% often have been angered because of things that happened that were outside of their control.
- In the last month, 43% often have felt difficulties were piling up so high that they could not overcome them.

**Figure 2.**
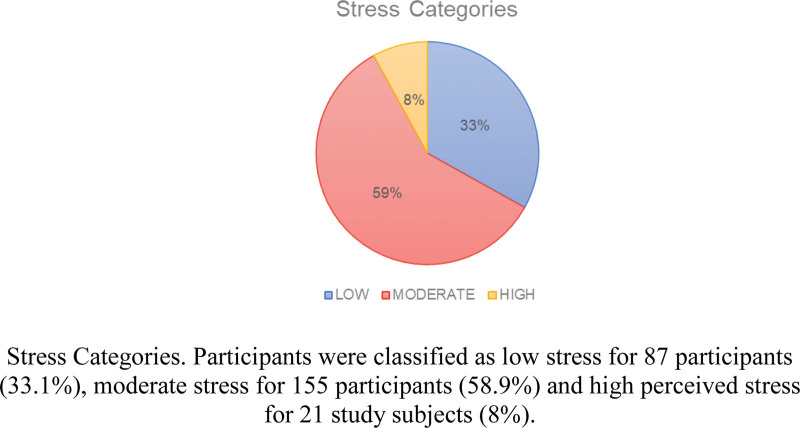
Stress categories.

In Table 5, individual scores on the PSS-10 ranged from 0 to 40 with higher scores indicating higher perceived stress (Never = 0, Almost never = 1, Sometimes = 2, Fairly often = 3, Very often = 4). The scores of the participants were classified into 3 categories based on their stress levels. Scores ranging from 0 to 13 were classified as low stress for 87 participants (33.1%). Scores ranging from 14 to 26 were classified as moderate stress for 155 participants (58.9%). Finally, scores ranging from 27 to 40 were classified as high perceived stress for 21 study subjects (8%) (Fig. 2).

**Table 5:** PSS Questionnaire’s scoring (n = 263).

Stress categories	N (%)	Minimum score	Maximum score	Mean	SD
Low	87 (33.1%)	5	10	7.5	2.7
Moderate	**155 (58.9%**)	15	25	20	3.8
High	21 (8%)	30	35	32.5	5.5

Numbers (%), Mean.

PSS-10 = Perceived Stress Scale (10-item questionnaire).

- Correlations were estimated to test the associations between study variables in subgroups of current participants.
  (1) Based on the aforementioned analysis, certain variables were chosen, and their correlations were established in the main age category (18–40 years old, n = 236) as the subgroup of all participants (n = 263). These variables include elevated BMI, increased WC, elevated waist-to-hip ratio, reduced sleep duration, diminished sleep efficiency and quality, moderate levels of PA, heightened intake of sugary foods, frequent consumption of fast meals, and moderate levels of perceived stress (Supplementary Material S7, Supplemental Digital Content, http://links.lww.com/MD/N80).

Weight parameters (BMI, WC, and waist/hip ratio) were significantly positively correlated to each other, and fast food while both BMI and WC were positively correlated to sleep quality and negatively to physical exercise (*P* < .05). BMI additionally was correlated positively to sleep duration, stress, and sugary food (*P* < .05). Sleep parameters were also correlated positively to each other and to stress (*P* < .05). PA was additionally significantly negatively correlated to stress. All data are tabulated in Supplementary Material S7, Supplemental Digital Content, http://links.lww.com/MD/N80.

(2) According to the data provided in Table 1, it can be observed that the students were categorized into several levels of medical study, namely Phase 1, Phase 2, and Phase 3, comprising 79.5% (n = 209) of the whole sample. As a result, a comprehensive analysis was undertaken to determine the correlations between the aforementioned variables and the various stages of educational attainment, as presented in Supplementary Material 8, Supplemental Digital Content, http://links.lww.com/MD/N81.

In phase 1 students, all weight parameters were positively correlated to each other; however, only BMI was correlated negatively to physical exercise (*P* < .05). All sleep parameters were positively correlated to each other and moderate levels of stress (*P* < .05). Additionally, moderate levels of exercise were negatively associated with stress (*P* < .05).

In phase 2 students, all weight parameters were positively correlated to each other; however, only BMI was correlated negatively to physical exercise (*P* < .05). However, sleep parameters were only correlated to each other without any significant correlations to other parameters. Also, other parameters did not show any significant correlations except the previously mentioned ones.

In phase 3 students, weight parameters were significantly correlated to each other and positively to fast meals. Also, both BMI and WC were correlated positively to sleep quality and negatively to PA (*P* < .05). BMI alone was additionally correlated positively to sleep duration, stress, and sugary food, while it negatively correlated to PA (*P* < .05). Sleep parameters were also correlated to each other and moderate levels of stress (*P* < .05). Additionally, moderate levels of stress were correlated positively with sugary food and negatively with moderate levels of PA (*P* < .05).

(3) Based on the data presented in Table 1, it was seen that 49 research participants, accounting for 18.6% of the total sample, were diagnosed with anxiety disorder. Consequently, an examination was conducted to establish the associations between the aforementioned indicators and the presence of anxiety disorder, as outlined in Table 6. The findings from the correlation analysis revealed a statistically significant positive association between high BMI and anxiety disorder (*R* = 0.54, *P* = .04). Conversely, sleep duration, efficiency, and quality; as well as moderate levels of PA were found to be negatively correlated with anxiety disorder (*P* < .05). A summary of all data is presented in Table 6.

**Table 6:** The correlations between different variables in anxiety disorder (25–40 years old, n = 49).

Parameter	Anxiety disorder	
	*r*	*P*
BMI = overweight (25–29.9)	**0.54**	**.04**
WC > 88	0.33	>.05
Waist/hip ratio > 0.85	0.31	>.05
Sleep duration	−0.58	.03
Sleep efficiency	−0.59	.03
Sleep quality	−0.65	.02
Moderate levels of physical activity	−0.53	.03
Moderate levels of perceived stress	**0.68**	**.02**
Intake of sugary foods	0.21	>.05
Consumption of fast meals	0.22	>.05

Pearson coefficient correlation analysis. The bold indicates the positive significant correlations whereas the red indicates the negative correlations. Statistical significance attributed to results with *P* < .05.

BMI = body mass index, WC = waist circumference.

## 4. Discussion:

In the studied group (n = 263), 44.5% took 30 to 60 minutes to fall asleep, and 51.7% reported fairly poor sleep quality. The mean sleep quality score was 2.6 ± 1.1, with a global PSQI score of 6 ± 2.1, indicating inadequate sleep quality for scores above 5.3, suggesting a widespread occurrence of sleep-related issues. Our findings aligned with another study that utilized the PSQI scale to assess sleep quality. This consistency in patterns across studies underscores the significance of the observed sleep problems and emphasizes the need for further attention to this issue.^[21]^ For example, similar proportions of those with prolonged sleep start latency and poor sleep quality were found in another study that looked at a similar cohort.^[22]^ Their results supported the idea that there were common difficulties with sleep duration and efficiency across a range of demographics, which was consistent with what we found in our study group. This agreement with other studies highlights the universality of sleep problems across many demographic groups and underscores the worldwide significance of these difficulties.^[23]^ Additional research emphasized the consequences of insufficient sleep efficiency, pointing out its links to a range of health problems, such as a higher chance of developing chronic illnesses and mental health difficulties.^[24]^ These associations verify the relevance of addressing sleep-related issues, underlining the necessity for focused treatments to promote sleep quality and general well-being in afflicted groups.

Regarding WC and sleep disorders, emerging research indicated a correlation between increased abdominal adiposity, as reflected in WC, and elevated risks of sleep disturbances, notably sleep apnea. Excessive abdominal fat can lead to alterations in respiratory function during sleep, contributing to breathing complications and disruptions in sleep patterns.^[25]^ This was in line with our findings of significant correlations between sleep parameters and weight parameters which highlighted the potential significance of lifestyle modifications, including weight management, in mitigating sleep-related concerns, particularly in individuals with increased WC. This association further underscores the critical need to address sleep-related concerns, emphasizing the urgency for targeted interventions aimed at improving sleep quality for enhanced overall well-being.^[26]^ Anxiety disorders contributed to insomnia and disrupted sleep patterns; while poor sleep, quality exacerbated symptoms of anxiety which was in line with our results of the presence of significant correlations between stress and sleep parameters. Literature suggested that managing anxiety could positively impact sleep outcomes, emphasizing integrated approaches in addressing both conditions effectively.^[27]^

The correlation analysis among different variables revealed insightful connections within the studied cohort aged between 18 and 40 years old. Specifically, examining the relationship between moderate levels of PA and moderate levels of perceived stress, the correlation coefficient stands at −0.57 which indicates a negative relationship between engaging in moderate PA and experiencing moderate levels of perceived stress.

Additionally, exploring the association between a waist/hip ratio > 0.85 and a WC > 88, the correlation coefficient is 0.32.^[28]^ The results of our study, however, indicated a more positive link between having a greater waist/hip ratio and having a WC surpassing 88 centimeters within our cohort, with a correlation value of 0.65 found between a waist/hip ratio > 0.85 and a WC > 88. These varying correlation strengths show how these variables are associated differently in various research groups, which may be due to varied lifestyles, demographics, or other underlying reasons among the cohorts under investigation.

In another study, when the relationship between moderate PA, perceived stress, waist/hip ratio (>0.85), and WC (>88) was examined in medical students at different stages, different results were obtained.^[28]^ Contrary to these findings, another research found no statistically significant link between the waist/hip ratio (>0.85) and moderate PA, despite our Phase 3 observations suggesting a negative correlation between the 2.^[29]^ Similar to how our study deviated from other research’s findings by not finding a significant link between perceived stress levels in Phase 1 and either the waist/hip ratio (>0.85) or the WC (>88).^[30]^ The results of our study’s phase 2 analysis confirmed that there were no meaningful relationships found between moderate PA and perceived stress levels and either the waist/hip ratio (>0.85) or the WC (>88). Though this was not seen in other research, Phase 3 showed a notable negative connection (>0.85) between moderate PA and the waist/hip ratio. These differences in correlations highlight the need for more research to confirm and fully explain these associations.

These findings suggest that the relationship between moderate PA, perceived stress, and measures of central obesity (waist/hip ratio and WC) varies across different phases among medical students. While moderate PA appeared to be related to central obesity in Phase 1 and Phase 3 based on waist/hip ratio, perceived stress did not show consistent associations with these anthropometric measures across the 3 phases.

Outlines participants’ responses to vigorous, moderate, and walking activities in the past 7 days. Notably, 34.6% engaged in vigorous activities, 68.6% in moderate activities, and 77.9% in walking activities. The computation of total PA MET-min/wk, combining these activities, revealed that the subjects collectively achieved 1200 MET of task minutes each week as moderate-intensity activities.

For example, their results were consistent with ours in that they showed that a sizable proportion of participants participated in physically demanding activities, even if a sizable majority of them did not indicate how long they participated.^[31]^

Table 5 categorizes participants into low, moderate-, and high-activity groups based on specific criteria. For example, the high activity category includes individuals engaging in ≥3 days of vigorous activity for at least 1500 min/wk. The study illustrates the distribution of participants across these categories, emphasizing percentages within each. It’s noteworthy that a recent study used different metrics to categorize activity levels, defining high activity as participating in strenuous activities for at least 5 days a week for a total of 1500 minutes.^[32]^ The categorization into low, moderate, and high activity levels provides insights into participants’ compliance with specified activity thresholds, offering a nuanced understanding of their activity patterns and adherence to recommended guidelines for optimal health benefits.

Another research initiative focused on assessing PA levels in middle-aged adults.^[33]^ Another research emphasized the limitations of self-reported data in accurately capturing PA levels.^[34]^ The authors acknowledged the need for more objective measures to complement self-reports and provide a clearer understanding of individuals’ activity patterns.^[35]^ If utilizing this metric in our research may assist in mitigating certain constraints associated with self-reported PA information. Augmenting traditional questionnaires with the objective measurement of PA levels, achieved through wearable devices or accelerometers, would yield a more precise insight into the authentic activity patterns within our study population. This approach could enhance the interpretation of self-reported WHO-5 Well-being Index scores and offer valuable insights into the interplay between PA, mental health, and overall well-being.

There is a troubling trend of excessive consumption of sweets, fast food, and snacks among various populations in both developed and developing countries which is attributed to the availability of these options in modern diets.^[36]^ Diets high in processed foods and added sugars are linked to an increased risk of obesity, cardiovascular disease, and metabolic disorders which was in line with our results of a positive correlation between weight parameters and eating fast food in women between 18 to 40 years and phase 3 students.^[37]^ Also, regular consumption of sweetened beverages such as soft drinks and juices is consistent across multiple studies worldwide. Because of the high sugar content and low nutritional value of these beverages, this habit is associated with an increased risk of weight gain, type 2 diabetes, and dental issues which emphasizes the importance of reducing sugary beverage consumption and promoting healthier alternatives such as water or natural fruit juices.^[38]^ Unfortunately, the common dietary pattern in various populations is a low frequency of consuming fruits, vegetables, and nutrient-rich foods.^[39,40]^ Studies emphasize the importance of eating fruits and vegetables for their essential vitamins, minerals, and fiber content, which play an important role in overall health and lowering the risk of chronic diseases like cancer, diabetes, and heart disease.^[41–44]^ We did not find a significant correlation between eating fast or sugary food and stress or sleep quality; however, increased weight parameters resulting from bad food habits were correlated to stress and sleep quality which can be explained by the association between stress and bad eating habits which increases the risk of obesity which highlight the importance of controlling stress to control healthy diet.^[45]^ Physical exercise can improve weight parameters as we found and can improve stress; however, this is difficult in obese persons as Brockmann 2020 et al found that increasing physical exercise by 1 hour per week was associated with a slight decrease in stress which made them recommend using another intervention to encourage the obese patient to increase PA and handle stress.^[46]^

In Saudi Arabia, there is a trend of eating unhealthy diets among female university students. A significant proportion of participants reported eating high-sugar and high-fat foods such as sweets (41.4% consuming 6 times per week) and fried foods (38.4% consuming 5 times per week) regularly.^[47]^ Similar findings were found in the consumption of sweetened beverages, with 35.7% reporting 4 times per week consumption.^[48]^ A considerable percentage (29.3%) of individuals regularly incorporated sugar or honey into their beverages, suggesting a pervasive utilization of added sugars while vegetables, fruits, and salads were consumed infrequently, with only a minority consuming them more than once per week.^[49]^ Dietary habits among university students in Kuwait and Dubai can be compared through studies exploring similarities or differences. Investigating the prevalence of high-sugar and high-fat food consumption among female university students in these regions could be a focus. For example, studies could reveal that a certain percentage, such as 60%, of female university students in both Kuwait and Dubai, commonly consumed high-sugar and high-fat foods.^[50]^ They could also investigate the frequency of eating out or drinking sugary beverages, drawing parallels or discrepancies with the Saudi study’s findings. Comparing these studies would reveal whether these dietary habits are consistent among female university students across these Arabic countries. Understanding the cultural and societal influences on dietary choices, particularly the preference for soft drinks and specific food preferences, may aid in understanding broader behavioral patterns associated with food consumption among female university students in these regions.

The average score of the WHO-5 Well-being Index in our sample was 7.8, with a standard deviation of 5.7 which indicated low scores related to poor well-being and potential depression in some participants based on the cutoff scores for the WHO-5. A study of 599 Saudi dental students found 42.1% were females and their mean WHO-5 score was 2.67 ± 0.94, which was lower than our results sample that indicated potential well-being issues among university students, despite variations by academic year being insignificant in that study. Comparisons show well-being levels may differ between educational institutions, student populations, environment, workload, and experiences could influence scores. More research is needed to understand determinants in these settings and develop effective well-being interventions for students and staff. The WHO-5 provided insight but more investigation is still required to clarify influencing variables and design strategies to enhance well-being in academic populations.^[51]^

Another study of women mostly young adults in Saudi Arabia found that over 40% reported ill-being or likely depression on the WHO-5 Well-Being Index.^[52]^ The proportion of ill-being/depression was significantly higher among those who lived in unfavorable environments, had morbidities, or experienced violence. This suggests external and health-related stressors may negatively impact well-being highlighting the detrimental effects of adverse life circumstances and health issues on Saudi women’s mental wellness.^[52]^ In conclusion, the studies demonstrate how well-being varies across populations based on demographic, environmental, and health-related factors, highlighting the need for further research to better understand determinants of wellness and to develop targeted interventions addressing stressors such as violence, living conditions, and disease burden.

Our investigation using the PSS revealed that a majority of respondents frequently experienced stress, as indicated by responses falling into the categories of “sometimes,” “often,” and “very often.” The prevalence of stress demonstrated variability across the questions, with a minimum percentage of 61.2% (Question 1) and a maximum of 79.7% (Question 6). Additionally, 59% of participants reported scores indicative of moderate stress, while 8% reported high stress levels. These figures aligned closely with a study conducted in Saudi Arabia by Abdel Rahman AG et al in 2013,^[53]^ where the prevalence of stress among medical students was reported to be 53%. Notably, this research found that gender did not significantly impact perceived stress, but interestingly, medical students who had confidants to share their stresses and concerns with others, such as close friends, demonstrated a notable statistical correlation between stressed and unstressed students. In another study conducted at King Saud University by Abdulghani HM et al in 2011,^[54]^ reported an overall stress prevalence of 63%, with 25% experiencing severe stress. Notably, stress levels were significantly high among females (75.7%).

In a study conducted in Ajman in 2011,^[55]^ the incidence of psychological morbidity among female students was (37%). Students enrolled in the MBBS program exhibited the highest levels of psychological morbidity, followed by those in Physiotherapy, Dentistry, and Pharmacy programs. Among the various stressors reported by students, concerns about the future and elevated parental expectations emerged as prominent contributors to psychological distress. Conversely, a research study carried out in Oman by Al Shamli et al in 2021^[56]^ observed that 51.4% of participants scored within the perceived stress threshold. Multivariate analysis indicated significant associations between perceived stress and factors such as age, years of study, and poor sleep quality with disrupted patterns.^[56]^

Our results fall in the range of the international results as suggested by this comprehensive systematic review by Fares J et al in 2016.^[57]^ This study encompassing 23 global studies, indicated considerable variability in stress levels among distinct samples of preclinical medical students, ranging from 20.9% to over 90%. Concurrently, prevalence rates for burnout and stress ranged from 27% to 75%. The observed variations were attributed to factors such as excessive workload, intense academic pressures necessitating rapid learning, and a profound emotional commitment to pursuing medicine as a future career. For our sample, causes of stress could be related to PA, in both phases 1 and 3, PA was found mildly protective against stress, *r* = −0.6 and −0.59 respectively, with *P* value < .05 in both phases. This was aligned with a clinical trial by van der Zwan^[58]^ aimed at decreasing stress among college students, where they found that PA played a protective role in their coping mechanisms. It is appropriate to assert that global events and the specific country under consideration have significantly influenced the prevalence of stress among medical students or physicians. An illustrative instance is the notable escalation in stress levels observed during the coronavirus disease 2019 pandemic, where stress reached a peak of 85.5%.^[59]^

### 4.1. Limitation

It is a cross-sectional design, so it cannot establish a cause-and-effect relationship or analyze behavior over time. the generalizability of this study might be limited due to the collection of samples from a single university. In addition, the number of students enrolled in medical colleges was minimal which did not allow the study to include an equal proportion of male students because of logistic barriers.

## 5. Conclusion

Adequate sleep quality is imperative for overall well-being, as reflected in the PSQI results, where 51.7%. The correlation between increased WC and sleep disorders underscores the importance of lifestyle modifications, particularly weight management, in mitigating sleep-related concerns. Recognizing the universality of sleep problems and their associations with health issues emphasizes the need for focused treatments to promote sleep quality and overall well-being.

In terms of PA, while 46% of participants met recommended levels, variations in categorization metrics highlight the need for standardized guidelines. Objective measures, like wearable devices, could offer a more accurate assessment of activity patterns. Correlation analysis reveals that the relationship between moderate PA, perceived stress, and central obesity measures varies across different phases among medical students. Understanding and addressing the nuances of PA patterns, stress levels, and anthropometric measures are essential for developing effective interventions tailored to specific phases of medical education.

The high prevalence of unhealthy dietary habits, such as frequent consumption of sweets and fried foods, underscores the need for targeted nutritional interventions among university students. Comparisons with studies in other Arabic countries clarified cultural and societal influences on dietary choices, aiding in understanding broader behavioral patterns associated with food consumption. Addressing the cultural context and promoting healthier alternatives can contribute to improving dietary habits and overall health among university students.

The WHO-5 Well-Being Index highlights potential well-being issues among university students, emphasizing the need for effective solutions by understanding influencing variables, clarifying determinants in academic settings, and designing targeted interventions to enhance well-being among students and staff.

Over 40% reporting stress-related issues underscores the prevalence of stress among participants. The correlation analysis between different variables and stress levels provides valuable insights into the complex interplay between lifestyle factors and perceived stress. The classification into low-, moderate, and high-stress categories underscores the need for individualized stress management approaches, considering the varied stress levels among participants.

Finally, the university and health authorities should provide additional awareness programs about the importance of a healthy lifestyle in improving health outcomes and decreasing the burden of chronic disease in the future.

In conclusion, this comprehensive analysis emphasizes the interconnectedness of sleep, PA, diet, emotional well-being, and stress among university students. Tailored interventions addressing specific aspects within each domain are essential for promoting holistic well-being in this population.

## Acknowledgments

We acknowledge all student and staff contributions and all the participants in this study. Also, we are thankful to our medical students who helped in the data collection Fatema Abdulkarim Saleh, Manal Ayesh Alanazi, Alaa Mohammad Seedi Mohammad, Taif Abdulwhaab Alahmadi, and Janna Fayez Alhejaili.

## Author contributions

**Conceptualization:** Amal M. Qasem Surrati.

**Data curation:** Eilaf Hasan Altayeb, Rahaf Mustafa Aljohani, Haya Shaher Ali Altouri, Rahaf Abdullah Alhawsawi.

**Investigation:** Wedyan Ali Almohammadi, Rahaf Mustafa Aljohani.

**Methodology:** Amal M. Qasem Surrati, Wedyan Ali Almohammadi, Rahaf Mustafa Aljohani, Rahaf Abdullah Alhawsawi.

**Project administration:** Eilaf Hasan Altayeb.

**Supervision:** Amal M. Qasem Surrati.

**Validation:** Amal M. Qasem Surrati.

**Writing – original draft:** Wedyan Ali Almohammadi, Rahaf Mustafa Aljohani, Haya Shaher Ali Altouri, Rahaf Abdullah Alhawsawi.

**Writing – review & editing:** Amal M. Qasem Surrati.

## Supplementary Material
