# Supplementary material for: The measured healthy lifestyle habits among Saudi university females in Medina, Saudi Arabia: A cross-sectional study
Source: Medicine (Baltimore). 2024 Jul 5;103(27):e38712. doi: 10.1097/MD.0000000000038712 (PMC11224813; doi:10.1097/MD.0000000000038712)
Supplement: Supplementary file 1 [file medi-103-e38712-s001.docx]

**Substance Abuse:**

Addiction is often seen as a detrimental pattern of behavior that negatively impacts one's overall well-being. The utilization of tobacco and other drugs has been associated with a multitude of adverse effects, including but not limited to cardiovascular disease, asthma, cancer, and brain harm. According to the findings obtained from our tobacco usage questionnaire, it was shown that the majority of our participants, namely 95%, identified as non-smokers. Additionally, a small percentage of 1.9% were classified as former smokers, while 2.3% were categorized as current smokers **Supplement 6**

.

| **Supplement 6**  **: Smoking use Questionnaire’s response (n=263)** | |
| --- | --- |
| **Questionnaire** | **Responses** |
| 1. Tobacco smoking | - **Non-smoker= 252(95.8%)** - Former smoker= 5(1.9%) - Current smoker= 6(2.3%) |
| 1. How much do you smoke (Current smoker=6)? | - Occasionally, less than 1 per day=2(33.3%) - 2-10/ day=4(66.7%) |
| 1. How long ago did you quit smoking (Former smoker=5)? | - Do not know= 4(80%) - < 6 month= 1(20%) |
| *Numbers (%) are shown.* | |
