# Supplementary material for: The measured healthy lifestyle habits among Saudi university females in Medina, Saudi Arabia: A cross-sectional study
Source: Medicine (Baltimore). 2024 Jul 5;103(27):e38712. doi: 10.1097/MD.0000000000038712 (PMC11224813; doi:10.1097/MD.0000000000038712)
Supplement: Supplementary file 2 [file medi-103-e38712-s002.docx]

**PSQI Questionnaire related to quality of sleep:**

The Pittsburgh Sleep Quality Index (PSQI) **comprises a set of 19 items that individuals self-rate**. **(Supplement 1**)

The scoring process only takes into account questions that are self-rated. The 19 elements that participants assessed themselves on **are aggregated to create seven distinct scores**, referred to as "components," with a scoring **range of 0 to 3 points.** Our results indicated that 44.5% needed 30 to 60 minutes to fall sleep and 51.7% described their sleep quality was fairly bad.

| **Supplement 1 :** **The Pittsburgh Sleep Quality Index (PSQI) self-rated questions** | | | | |
| --- | --- | --- | --- | --- |
| **Self-Rated Questions** | **Overall response (n=263)** | | | |
| ***Scoring*** | ***0*** | ***1*** | ***2*** | ***3*** |
| 1. During the past month, what time have you usually gone to bed at night | *> 7 hours*  20(7.6%) | *6-7 hours*  40(15.2%) | *5-6 hours*  **160(60.8%)** | *< 5 hours*  43(16.3%) |
| 1. During the past month, how long (in minutes) has it usually taken you to fall asleep each | *≤ 15 minutes*  20(7.6%) | *16-30 minutes*  86(32.7%) | *31-60 minutes*  **117(44.5%)** | *> 60 minutes*  40(15.2%) |
| 1. During the past month, what time have you usually gotten up in the morning* | 7±3.6 | | | |
| 1. During the past month, how many hours of actual sleep did you get at night* | 7.5±2.7 | | | |
| 1. During the past month, how often have you had trouble sleeping because you : | | | | |
|  | *Three or more times a week* | *Once or twice a week* | *Less than once a week* | *Not during the past month* |
| 1. Cannot get to sleep within 30 minutes | 55(20.9%) | 49(18.6%) | 57(21.7%) | 102(38.8%) |
| 1. Wake up in the middle of the night or early morning | 40(15.2%) | 45(17.1%) | 78(29.7%) | 100(38%) |
| 1. Have to get up to use the bathroom | 24(9.1%) | 50(19%) | 59(22.4%) | 130(49.4%) |
| 1. Cannot breathe comfortably | 14(5.3%) | 22(8.4%) | 33(12.5%) | 194(73.8%) |
| 1. Cough or snore loudly | 11(4.2%) | 14(5.3%) | 15(5.7%) | 223(84.8%) |
| 1. Feel too cold | 23(8.7%) | 28(10.6%) | 26(9.9%) | 186(70.7%) |
| 1. Feel too hot | 23(8.7%) | 52(19.8%) | 70(26.6%) | 118(44.9%) |
| 1. Have bad dreams | 10(3.8%) | 20(7.6%) | 43(16.3%) | 190(72.2%) |
| 1. Have pain | 55(20.9%) | 49(18.6%) | 57(21.7%) | 102(38.8%) |
| 1. During the past month, how would you rate your sleep quality overall? | *Very good*  55(20.9%) | *Fairly good*  55(20.9%) | *Fairly bad*  **136(51.7%)** | *Very bad*  17(6.5%) |
| 1. During the past month, how often have you taken medicine to help you sleep (prescribed or over the counter)? | *Not during the past month*  217(82.5%) | *Less than once a week*  22(8.4%) | *Once or twice*  *a week*  15(5.7%) | *Three or more times a week*  9(3.4%) |
| 1. During the past month, how often have you had trouble staying awake while driving, eating meals, or engaging in social activity? | *Not during the past month*  167(63.5%) | *Less than once a week*  54(20.5%) | *Once or twice*  *a week*  27(10.3%) | *Three or more times a week*  15(5.7%) |
| 1. During the past month, how much of a problem has it been for you to keep up enough enthusiasm to get things done? | *No problem at all*  81(30.8%) | *Only a very slight problem*  82(31.2%) | *Somewhat of a problem*  80(30.4%) | *A very big problem*  20(7.6%) |
| *Number (%) are shown. *Mean ± SD (standard deviation)* | | | | |

Seven components are equally weighted on the Pittsburgh Sleep Quality Index (PSQI) questionnaire's nineteen items: **subjective sleep quality** (one item), **sleep latency** (two items), **sleep duration** (one item), **sleep efficiency** (three items), **sleep disturbances** (nine items), **daytime dysfunction** (two items), and **sedative use** (one item). In every instance, a score **of "0" signifies the absence of difficulty**, whereas a score of **"3" signifies the presence of significant difficulty**. In our subjects, the data indicated poor overall sleep duration and efficiency among participants with **relative difficulty (2.6±1.1) according to sleep quality**.

The summation of the seven component scores results in a single **"global" score**, which **spans from 0 to 19 points**. A score of **0 signifies the absence of any issues**, while a score of **19 indicates serious difficulties across all categories (Table 2)**. Also, **the global PSQI** score was derived by **summing the values of the seven components**, which comprised the scale's conceivable **range of scores from 0 to 19**. A global score greater than five signifies an inadequate SQ (6±2.1) that was determined in our subjects with 45.2% sleep efficiency, sleep efficiency of less than 80% was thought to be reduced sleep efficiency (Normal sleep efficiency is considered to be 80% or greater, *Lin YQ, Lin ZX, Wu YX, Wang L, Zeng ZN, Chen QY, Wang L, Xie XL, Wei SC. Reduced Sleep Duration and Sleep Efficiency Were Independently Associated With Frequent Nightmares in Chinese Frontline Medical Workers During the Coronavirus Disease 2019 Outbreak. Front Neurosci. 2021 Jan 20;14:631025*.) (**Table 2**).
