# Supplementary material for: The measured healthy lifestyle habits among Saudi university females in Medina, Saudi Arabia: A cross-sectional study
Source: Medicine (Baltimore). 2024 Jul 5;103(27):e38712. doi: 10.1097/MD.0000000000038712 (PMC11224813; doi:10.1097/MD.0000000000038712)
Supplement: Supplementary file 3 [file medi-103-e38712-s003.docx]

**IPAQ- TM Questionnaire related to quality of exercise.**

The PA (Physical activity) categories of the sample are presented in Table 4, according to the evaluating procedure of the International Physical Activity Questionnaire (IPAQ). In all, 91 individuals, accounting for 34.6% of the sample, engaged in vigorous physical activities, whereas the other 172 participants, constituting 65.4% of the total sample size of 263, did not partake in such activities. Among the sample of 172 participants, a majority of 118 individuals (68.6%) engaged in moderate physical activity. Among the total sample size of 263 participants, a significant majority of 205 individuals (77.9%) engaged in walking activities for a minimum duration of 10 minutes. In aggregate, the subjects achieved a total of 1200 MET of task-minutes each week as Moderate-intensity activities indicator (**Supplement 2**).

According to Hallal et al., a total physical activity score was computed as

Walking MET-minutes/week = 3.3 * walking minutes * walking days

Moderate MET-minutes/week = 4.0 * moderate-intensity activity minutes * moderate days

Vigorous MET-minutes/week = 8.0 * vigorous-intensity activity minutes * vigorous-intensity days

Total physical activity MET-minutes/week = sum of Walking + Moderate + Vigorous MET

minutes/week scores. *Hallal PC, Victora CG, Wells JC, Lima RC. Physical inactivity: prevalence and associated variables in Brazilian adults. Med Sci Sports Exerc 2003;35:1894e900.*

| **Supplement 2 : IPAQ- TM Questionnaire’s response.** | | |
| --- | --- | --- |
| **Questionnaire** | **Responses** | |
| 1. During the last 7 days, how many days did you do vigorous physical activities like heavy lifting, digging, aerobics, or fast bicycling? **(n=263)** | *Days per week*  91(34.6%) | *No*  172(65.4%) |
| 1. How much time did you usually spend doing vigorous physical activities on one of those days? **(n=91)** | *Minutes per day*  38(41.8%) | *Don’t know*.  53(58.2%) |
| 1. During the last 7 days, on how many days did you do moderate physical activities like carrying light loads, bicycling at a regular pace, or doubles tennis? Do not include walking. **(n=172)** | *Days per week*  118(68.6%) | *No*  54(31.4%) |
| 1. How much time did you usually spend doing moderate physical activities on one of those days? **(n=118)** | *Minutes per day*  66(55.9%) | *Don’t know.*  52(44.1%) |
| 1. During the last 7 days, on how many days did you walk for at least 10 minutes at a time? **(n=263)** | *Days per week*  **205(77.9%)** | *No*  58(22.1%) |
| 1. How much time did you usually spend walking on one of those days? **(n=205)** | *Minutes per day*  166(81%) | *Don’t know*.  39(19%) |
| 1. During the last 7 days, how much time did you spend sitting on a weekday? **(n=263)** | *Minutes per day*  **204(77.6%)** | *Don’t know.*  52(22.4%) |
| ***^#^***Vigorous Activity | 300±111 MET-min/week | |
| ***^#^***Moderate Activity | 500±50.9 MET-min/week | |
| ***^#^***Walking Activity | 400±80.3 MET-min/week | |
| **Total MET-min/week** | **1200 MET-min/week** (**Moderate-intensity activities)** | |
| *Numbers (%) are shown.* ***^#^Mean ±SD*** *(standard deviation).* *International Physical Activity Questionnaires (IPAQ)* | | |

In general, 46% of the participants achieved the recommended level of at least 500 minutes per week of moderate physical activity for 5 days, which is considered a reasonable threshold for obtaining health benefits (**supplement 2, Figure 1**).
