# Supplementary material for: The measured healthy lifestyle habits among Saudi university females in Medina, Saudi Arabia: A cross-sectional study
Source: Medicine (Baltimore). 2024 Jul 5;103(27):e38712. doi: 10.1097/MD.0000000000038712 (PMC11224813; doi:10.1097/MD.0000000000038712)
Supplement: Supplementary file 4 [file medi-103-e38712-s004.docx]

- **Questionnaire related to quality of diet.**

**Supplement 3** presents data on the high prevalence of frequent consumption of sweets, fast meals, and snacks, as well as low portions of fruits and vegetables per day among the participants. Additionally, it highlights the high percentage of participants who reported regular intake of sugar and honey in their tea, coffee, or other beverages.

| **Supplement 3: Quality of Diet Questionnaire’s response (n=263)** | | | | | |
| --- | --- | --- | --- | --- | --- |
| **Questionnaire** | **Responses** | | | | |
|  | *6 times* | *5 times* | *4 times* | *3 times* |  |
| 1. How often do you eat meals in a day (including tea, coffee, fruits, salads, snacks)? | 57(21.7%) | 59(22.4%) | 74(28.1%) | 73(27.8%) |  |
|  | *At least once daily* | *3 to 6 times a week* | *1 to 2 times a week* | *2 to 3 times a month* | *Once a month or less.* |
| 1. How often do you drink sweetened beverages like soft drinks, juices, etc.? | 55(20.9%) | 59(22.4%) | 94(35.7%) | 29(11%) | 26(9.9%) |
| 1. How often do you eat sweets such as Halwa, Kunafa, cookies, etc.? | **109(41.4%)** | 100(38%) | 26(9.9%) | 22(8.4%) | 6(2.3%) |
| 1. How often do you eat fried foods such as French fries, Chicken Strips, etc.? | 55(20.9%) | **101(38.4%)** | 56(21.3%) | 44(16.7%) | 7(2.7%) |
| 1. How often do you eat high salt snacks such as Chips, Nuts, etc.? | 43(16.3%) | **104(39.5%)** | 66(25.1%) | 44(16.7%) | 6(2.3%) |
| 1. How often do you consume sugar and honey in tea, coffee, etc? | 77(29.3%) | **78(29.7%)** | 55(20.9%) | 33(12.5%) | 20(7.6%) |
| 1. How often do you eat saturated fat like mutton fat, egg yolks, etc.? | 55(20.9%) | 54(20.5%) | 45(17.1%) | 54(20.5%) | 55(20.9%) |
| 1. How often do you eat refined food items like burgers, pizza, etc.? | 12(4.6%) | 57(21.7%) | **82(31.2%)** | 79(30%) | 33(12.5%) |
| 1. How often do you eat ghee, butter, cream, mayonnaise, etc.? | 77(29.3%) | 65(24.7%) | 75(28.5%) | 22(8.4%) | 24(9.1%) |
|  | | | | | |
|  | *Every time in the main diet* | *At least once a day* | *3 to 4 times a week* | *1 time a week* | *Less than once a week.* |
| 1. How often do you eat fruit and salad? | 6(2.3%) | 44(16.7%) | 66(25.1%) | **104(39.5%)** | 43(16.3%) |
| 1. How often do you eat sprouted pulses and green vegetables? | 12(4.6%) | 57(21.7%) | 79(30%) | **82(31.2%)** | 33(12.5%) |
|  | | | | | |
|  | *More than 3 times a week* | *More than once a week* | *2 times in a month* | *1 time in a month* |  |
| 1. How often do you eat out of the house (such as wedding, party, family function etc.)? | 12(4.6%) | 37(14%) | 131(49.8%) | 83(31.6%) |  |
| *Numbers (%) are shown.* | | | | | |
