# Supplementary material for: The measured healthy lifestyle habits among Saudi university females in Medina, Saudi Arabia: A cross-sectional study
Source: Medicine (Baltimore). 2024 Jul 5;103(27):e38712. doi: 10.1097/MD.0000000000038712 (PMC11224813; doi:10.1097/MD.0000000000038712)
Supplement: Supplementary file 5 [file medi-103-e38712-s005.docx]

**WHO (Five) Well-Being Index questionnaire related to quality of emotional well-being**

**Supplement 4**

displays the data indicating that a significant majority of the individuals experienced less than 50% of their time feeling pleasant, peaceful, active, and waking up feeling refreshed.

| **Supplement 4**  **: Well-Being Index Questionnaire’s response (n=263)** | | | | | |  |
| --- | --- | --- | --- | --- | --- | --- |
| **WHO-5** | **At no time** | **Some of the time** | **Less than half of the time** | **More than half of the time** | **Most of the time** | **All of the time** |
| I have felt cheerful and in good spirits | 12(4.6%) | 29(11%) | **83(31.6%)** | 54(20.5%) | 64(24.3%) | 21(8%) |
| I have felt calm and relaxed | 11(4.2%) | 37(14.1%) | **84(31.9%)** | 51(19.4%) | 66(25.1%) | 14(5.3%) |
| I have felt active and vigorous | 13(4.9%) | 39(14.8%) | **83(31.6%)** | 69(26.2%) | 50(19%) | 9(3.4%) |
| I woke up feeling fresh and rested | 20(7.6%) | 40(15.2%) | **68(25.9%)** | 63(24%) | 51(19.4%) | 21(7.9%) |
| My daily life has been filled with things that interested me | 30(11.4%) | 42(16%) | **73(27.8%)** | 67(25.5%) | 44(16.7%) | 7(2.7%) |
| *Numbers (%) are shown.* | | | | | |  |
