# Supplementary material for: The measured healthy lifestyle habits among Saudi university females in Medina, Saudi Arabia: A cross-sectional study
Source: Medicine (Baltimore). 2024 Jul 5;103(27):e38712. doi: 10.1097/MD.0000000000038712 (PMC11224813; doi:10.1097/MD.0000000000038712)
Supplement: Supplementary file 6 [file medi-103-e38712-s006.docx]

**PSS-10 questionnaire related to the quality of stress.**

**Supplement 5**

displays the item analysis conducted on the responses of the research participants for the Perceived Stress Scale (PSS). A significant proportion of our study participants, above 40%, reported experiencing sometimes of felt stress in their lives.

| **Supplement 5**  **PSS Questionnaire’s response (n=263)** | | | | | |
| --- | --- | --- | --- | --- | --- |
| **Questionnaire** | **Never** | **Almost never** | **Sometimes** | **Fairly often** | **Very often** |
| 1. In the last month, how often have you been upset because of something that happened unexpectedly? | 29(11%) | 73(27.8%) | **107(40.7%)** | 35(13.3%) | 19(7.2%) |
| 1. In the last month, how often have you felt that you were unable to control the important things in your life? | 24(9.1%) | 60(22.8%) | **112(42.6%)** | 44(16.7%) | 23(8.7%) |
| 1. In the last month, how often have you felt nervous and stressed? | 24(9.1%) | 42(16%) | **110(41.8%)** | 43(16.3%) | 44(16.7%) |
| 1. In the last month, how often have you felt confident about your ability to handle your personal problems? | 22(8.4%) | 37(14.1%) | **100(38%)** | 46(17.5%) | 58(22.1%) |
| 1. In the last month, how often have you felt that things were going your way? | 29(11%) | 38(14.4%) | 99(37.6%) | 53(20.2%) | 44(16.7%) |
| 1. In the last month, how often have you found that you could not cope with all the things that you had to do? | 19(7.2%) | 55(20.9%) | **122(46.4%)** | 33(12.5%) | 34(12.9%) |
| 1. In the last month, how often have you been able to control irritations in your life? | 30(11.4%) | 41(15.6%) | **111(42.2%)** | 55(20.9%) | 26(9.9%) |
| 1. In the last month, how often have you felt that you were on top of things? | 25(9.5%) | 44(16.7%) | **100(38%)** | 48(18.3%) | 46(17.5%) |
| 1. In the last month, how often have you been angered because of things that happened that were outside of your control? | 25(9.5%) | 25(9.5%) | **111(42.2%)** | 57(21.7%) | 45(17.1%) |
| 1. In the last month, how often have you felt difficulties were piling up so high that you could not overcome them? | 25(9.5%) | 22(8.4%) | **113(43%)** | 53(20.2%) | 50(19%) |
| *Numbers (%) are shown. PSS = Perceived Stress Scale.* | | | | | |
