# Supplementary material for: The measured healthy lifestyle habits among Saudi university females in Medina, Saudi Arabia: A cross-sectional study
Source: Medicine (Baltimore). 2024 Jul 5;103(27):e38712. doi: 10.1097/MD.0000000000038712 (PMC11224813; doi:10.1097/MD.0000000000038712)
Supplement: Supplementary file 7 [file medi-103-e38712-s007.docx]

**Correlations were estimated to test the associations between study variables in subgroups of current participants.**

Based on the aforementioned analysis, certain variables were chosen, and their correlations were established in the main age category (18-40 years old, n=236) as the subgroup of our all participants (n=263). These variables include elevated body mass index (BMI), increased waist circumference (WC), elevated waist-to-hip ratio, reduced sleep duration, diminished sleep efficiency and quality, moderate levels of physical activity, heightened intake of sugary foods, frequent consumption of fast meals, and moderate levels of perceived stress (**Supplement 7**

1. ). The result of the correlation analysis indicated that there was significantly positive relationships between BMI and WC, waist/hip ratio, sleep duration and quality (P < 0.05), between BMI and heightened intake of sugary foods, frequent consumption of fast meals, and moderate levels of perceived stress (P < 0.05) but high BMI was negatively correlated with Moderate levels of physical activity (r = -0.59, P= 0.04). All data are tabulated in **Supplement 7**.

| Supplement 7 : The correlations between different variables (18-40 years old) | | | | | | | | | | | | | | | | | | | | | |
| --- | --- | --- | --- | --- | --- | --- | --- | --- | --- | --- | --- | --- | --- | --- | --- | --- | --- | --- | --- | --- | --- |
| Parameter | BMI  Overweight (25 to 29.9) | | WC> 88 | | Waist/hip ratio>0.85 | | Sleep duration | | Sleep efficiency | | Sleep quality | | Moderate levels of physical activity | | Moderate levels of perceived stress | | Intake of sugary foods | | Consumption of fast meals | | |
|  | ***r*** | ***P*** | ***r*** | ***P*** | ***r*** | ***P*** | ***r*** | ***P*** | ***r*** | ***P*** | ***r*** | ***P*** | ***r*** | ***P*** | ***r*** | ***P*** | ***r*** | ***P*** | ***r*** | ***P*** |  |
| BMI  Overweight (25 to 29.9) | 1 | - | **0.62** | **0.03** | **0.55** | **0.03** | **0.52** | **0.05** | 0.33 | 0.06 | **0.57** | **0.04** | -0.59 | 0.04 | **0.58** | **0.03** | **0.76** | **0.01** | **0.66** | **0.01** |  |
| WC> 88 | **0.62** | **0.03** | 1 | - | **0.65** | **0.02** | 0.31 | >0.05 | 0.22 | >0.05 | **0.54** | **0.05** | -0.55 | 0.04 | 0.32 | >0.05 | 0.22 | >0.05 | **0.49** | **0.04** |  |
| Waist/hip ratio>0.85 | **0.55** | **0.03** | **0.65** | **0.02** | 1 | - | 0.30 | >0.05 | 0.21 | >0.05 | 0.32 | >0.05 | 0.13 | >0.05 | 0.24 | >0.05 | 0.26 | >0.05 | **0.55** | **0.04** |  |
| Sleep duration | **0.52** | **0.05** | 0.31 | >0.05 | 0.30 | >0.05 | 1 | - | **0.65** | **0.02** | **0.75** | **0.01** | 0.34 | >0.05 | **0.53** | **0.03** | 0.35 | >0.05 | 0.36 | >0.05 |  |
| Sleep efficiency | 0.33 | 0.06 | 0.22 | >0.05 | 0.21 | >0.05 | **0.65** | **0.02** | 1 | - | **0.85** | **0.01** | 0.38 | >0.05 | **0.55** | **0.04** | 0.38 | >0.05 | 0.39 | >0.05 |  |
| Sleep quality | **0.57** | **0.04** | **0.54** | **0.05** | 0.32 | >0.05 | **0.75** | **0.01** | **0.85** | **0.01** | 1 | - | 0.32 | >0.05 | **0.59** | **0.03** | 0.32 | >0.05 | 0.31 | >0.05 |  |
| Moderate levels of physical activity | -0.59 | 0.04 | -0.55 | 0.04 | 0.13 | >0.05 | 0.34 | >0.05 | 0.38 | >0.05 | 0.32 | >0.05 | 1 | - | -0.57 | 0.05 | 0.42 | >0.05 | 0.41 | >0.05 |  |
| Moderate levels of perceived stress | **0.58** | **0.03** | 0.32 | >0.05 | 0.24 | >0.05 | **0.53** | **0.03** | **0.55** | **0.04** | **0.59** | **0.03** | -0.57 | 0.05 | 1 | - | 0.22 | >0.05 | 0.21 | >0.05 |  |
| Intake of sugary foods | **0.76** | **0.01** | 0.22 | >0.05 | 0.26 | >0.05 | 0.35 | >0.05 | 0.38 | >0.05 | 0.32 | >0.05 | 0.42 | >0.05 | 0.22 | >0.05 | 1 | - | 0.42 | >0.05 |  |
| Consumption of fast meals | **0.66** | **0.01** | **0.49** | **0.04** | **0.55** | **0.04** | 0.36 | >0.05 | 0.39 | >0.05 | 0.31 | >0.05 | 0.41 | >0.05 | 0.21 | >0.05 | 0.42 | >0.05 | 1 | - |  |
| *Pearson coefficient correlation and* *Chi square test analysis. The bold indicates the positive significant correlations whereas the red indicates the negative correlations. Statistical significance attributed to results with p < 0.05.* | | | | | | | | | | | | | | | | | | | | | |
