# Supplementary material for: The measured healthy lifestyle habits among Saudi university females in Medina, Saudi Arabia: A cross-sectional study
Source: Medicine (Baltimore). 2024 Jul 5;103(27):e38712. doi: 10.1097/MD.0000000000038712 (PMC11224813; doi:10.1097/MD.0000000000038712)
Supplement: Supplementary file 8 [file medi-103-e38712-s008.docx]

1. According to the data provided in Table 1, it can be observed that the students were categorized into several levels of medical study, namely Phase 1, Phase 2, and Phase 3, comprising 79.5% (n=209) of the whole sample. As a result, a comprehensive analysis was undertaken to determine the correlations between the aforementioned variables and the various stages of educational attainment, as presented in Supplement 8

| Supplement 8: The correlations between different variables based on the medical students’ study levels (n=209) | | | | | | | | | | | | | | | | | | | | |
| --- | --- | --- | --- | --- | --- | --- | --- | --- | --- | --- | --- | --- | --- | --- | --- | --- | --- | --- | --- | --- |
| Phase 1 | | | | | | | | | | | | | | | | | | | | |
| Parameter | BMI  Overweight (25 to 29.9) | | WC> 88 | | Waist/hip ratio>0.85 | | Sleep duration | | Sleep efficiency | | Sleep quality | | Moderate levels of physical activity | | Moderate levels of perceived stress | | Intake of sugary foods | | Consumption of fast meals | |
|  | ***r*** | ***P*** | ***r*** | ***P*** | ***r*** | ***P*** | ***r*** | ***P*** | ***r*** | ***P*** | ***r*** | ***P*** | ***r*** | ***P*** | ***r*** | ***P*** | ***r*** | ***P*** | ***r*** | ***P*** |
| BMI  Overweight (25 to 29.9) | 1 | - | **0.61** | **0.03** | **0.54** | **0.03** | 0.32 | >0.05 | 0.31 | >0.05 | 0.37 | >0.05 | **-0.57** | **0.04** | 0.38 | >0.05 | 0.36 | >0.05 | 0.26 | >0.05 |
| WC> 88 | **0.61** | **0.03** | 1 | - | **0.66** | **0.02** | 0.32 | >0.05 | 0.21 | >0.05 | 0.24 | >0.05 | -0.25 | >0.05 | 0.30 | >0.05 | 0.21 | >0.05 | 0.39 | >0.05 |
| Waist/hip ratio>0.85 | **0.54** | **0.03** | **0.66** | **0.02** | 1 | - | 0.31 | >0.05 | 0.22 | >0.05 | 0.34 | >0.05 | 0.16 | >0.05 | 0.26 | >0.05 | 0.29 | >0.05 | 0.35 | >0.05 |
| Sleep duration | 0.32 | >0.05 | 0.32 | >0.05 | 0.31 | >0.05 | 1 | - | **0.66** | **0.02** | **0.76** | **0.01** | 0.32 | >0.05 | **0.52** | **0.03** | 0.31 | >0.05 | 0.32 | >0.05 |
| Sleep efficiency | 0.31 | >0.05 | 0.21 | >0.05 | 0.22 | >0.05 | **0.66** | **0.02** | 1 | - | **0.87** | **0.01** | 0.39 | >0.05 | **0.59** | **0.04** | 0.33 | >0.05 | 0.34 | >0.05 |
| Sleep quality | 0.37 | >0.05 | 0.24 | >0.05 | 0.34 | >0.05 | **0.76** | **0.01** | **0.87** | **0.01** | 1 | - | 0.34 | >0.05 | **0.60** | **0.03** | 0.31 | >0.05 | 0.33 | >0.05 |
| Moderate levels of physical activity | **-0.57** | **0.04** | -0.25 | >0.05 | 0.16 | >0.05 | 0.32 | >0.05 | 0.39 | >0.05 | 0.34 | >0.05 | 1 | - | -0.60 | 0.05 | 0.41 | >0.05 | 0.36 | >0.05 |
| Moderate levels of perceived stress | 0.38 | >0.05 | 0.30 | >0.05 | 0.26 | >0.05 | **0.52** | **0.03** | **0.59** | **0.04** | **0.60** | **0.03** | -0.60 | 0.05 | 1 | - | 0.23 | >0.05 | 0.18 | >0.05 |
| Intake of sugary foods | 0.36 | >0.05 | 0.21 | >0.05 | 0.29 | >0.05 | 0.31 | >0.05 | 0.33 | >0.05 | 0.31 | >0.05 | 0.41 | >0.05 | 0.23 | >0.05 | 1 | - | 0.41 | >0.05 |
| Consumption of fast meals | 0.26 | >0.05 | 0.21 | >0.05 | 0.35 | >0.05 | 0.32 | >0.05 | 0.34 | >0.05 | 0.33 | >0.05 | 0.36 | >0.05 | 0.18 | >0.05 | 0.41 | >0.05 | 1 | - |
| Phase 2 | | | | | | | | | | | | | | | | | | | | |
| Parameter | BMI  Overweight (25 to 29.9) | | WC> 88 | | Waist/hip ratio>0.85 | | Sleep duration | | Sleep efficiency | | Sleep quality | | Moderate levels of physical activity | | Moderate levels of perceived stress | | Intake of sugary foods | | Consumption of fast meals | |
|  | ***r*** | ***P*** | ***r*** | ***P*** | ***r*** | ***P*** | ***r*** | ***P*** | ***r*** | ***P*** | ***r*** | ***P*** | ***r*** | ***P*** | ***r*** | ***P*** | ***r*** | ***P*** | ***r*** | ***P*** |
| BMI  Overweight (25 to 29.9) | 1 | - | **0.51** | **0.04** | **0.44** | **0.04** | 0.31 | >0.05 | 0.33 | >0.05 | 0.39 | >0.05 | **-0.59** | **0.04** | 0.37 | >0.05 | 0.32 | >0.05 | 0.25 | >0.05 |
| WC> 88 | **0.51** | **0.04** | 1 | - | **0.60** | **0.02** | 0.30 | >0.05 | 0.23 | >0.05 | 0.25 | >0.05 | -0.27 | >0.05 | 0.34 | >0.05 | 0.26 | >0.05 | 0.31 | >0.05 |
| Waist/hip ratio>0.85 | **0.44** | **0.04** | **0.60** | **0.02** | 1 | - | 0.32 | >0.05 | 0.23 | >0.05 | 0.37 | >0.05 | 0.19 | >0.05 | 0.22 | >0.05 | 0.39 | >0.05 | 0.34 | >0.05 |
| Sleep duration | 0.31 | >0.05 | 0.30 | >0.05 | 0.32 | >0.05 | 1 | - | **0.67** | **0.02** | **0.76** | **0.01** | 0.32 | >0.05 | 0.33 | >0.05 | 0.34 | >0.05 | 0.33 | >0.05 |
| Sleep efficiency | 0.33 | >0.05 | 0.23 | >0.05 | 0.23 | >0.05 | **0.67** | **0.02** | 1 | - | **0.86** | **0.01** | 0.34 | >0.05 | 0.49 | >0.05 | 0.35 | >0.05 | 0.37 | >0.05 |
| Sleep quality | 0.39 | >0.05 | 0.25 | >0.05 | 0.37 | >0.05 | **0.76** | **0.01** | **0.87** | **0.01** | 1 | - | 0.36 | >0.05 | 0.40 | >0.05 | 0.36 | >0.05 | 0.37 | >0.05 |
| Moderate levels of physical activity | **-0.59** | **0.04** | -0.27 | >0.05 | 0.19 | >0.05 | 0.32 | >0.05 | 0.34 | >0.05 | 0.36 | >0.05 | 1 | - | -0.41 | >0.05 | 0.40 | >0.05 | 0.38 | >0.05 |
| Moderate levels of perceived stress | 0.37 | >0.05 | 0.34 | >0.05 | 0.22 | >0.05 | 0.33 | >0.05 | 0.49 | >0.05 | 0.40 | >0.05 | -0.41 | >0.05 | 1 | - | 0.21 | >0.05 | 0.16 | >0.05 |
| Intake of sugary foods | 0.32 | >0.05 | 0.26 | >0.05 | 0.39 | >0.05 | 0.34 | >0.05 | 0.35 | >0.05 | 0.36 | >0.05 | 0.40 | >0.05 | 0.21 | >0.05 | 1 | - | 0.43 | >0.05 |
| Consumption of fast meals | 0.25 | >0.05 | 0.31 | >0.05 | 0.34 | >0.05 | 0.33 | >0.05 | 0.37 | >0.05 | 0.37 | >0.05 | 0.38 | >0.05 | 0.16 | >0.05 | 0.43 | >0.05 | 1 | - |
| Phase 3 | | | | | | | | | | | | | | | | | | | | |
| Parameter | BMI  Overweight (25 to 29.9) | | WC> 88 | | Waist/hip ratio>0.85 | | Sleep duration | | Sleep efficiency | | Sleep quality | | Moderate levels of physical activity | | Moderate levels of perceived stress | | Intake of sugary foods | | Consumption of fast meals | |
|  | ***r*** | ***P*** | ***r*** | ***P*** | ***r*** | ***P*** | ***r*** | ***P*** | ***r*** | ***P*** | ***r*** | ***P*** | ***r*** | ***P*** | ***r*** | ***P*** | ***r*** | ***P*** | ***r*** | ***P*** |
| BMI  Overweight (25 to 29.9) | 1 | - | **0.61** | **0.03** | **0.58** | **0.03** | **0.59** | **0.05** | 0.37 | 0.06 | **0.59** | **0.04** | -0.66 | 0.04 | **0.59** | **0.03** | **0.76** | **0.01** | **0.67** | **0.01** |
| WC> 88 | **0.61** | **0.03** | 1 | - | **0.63** | **0.02** | 0.21 | >0.05 | 0.32 | >0.05 | **0.55** | **0.05** | -0.59 | 0.04 | 0.33 | >0.05 | 0.21 | >0.05 | **0.50** | **0.04** |
| Waist/hip ratio>0.85 | **0.58** | **0.03** | **0.63** | **0.02** | 1 | - | 0.32 | >0.05 | 0.22 | >0.05 | 0.31 | >0.05 | 0.16 | >0.05 | 0.25 | >0.05 | 0.27 | >0.05 | **0.59** | **0.04** |
| Sleep duration | **0.59** | **0.05** | 0.21 | >0.05 | 0.32 | >0.05 | 1 | - | **0.67** | **0.02** | **0.78** | **0.01** | 0.30 | >0.05 | **0.56** | **0.03** | 0.36 | >0.05 | 0.35 | >0.05 |
| Sleep efficiency | 0.37 | 0.06 | 0.32 | >0.05 | 0.22 | >0.05 | **0.67** | **0.02** | 1 | - | **0.82** | **0.01** | 0.34 | >0.05 | **0.57** | **0.04** | 0.33 | >0.05 | 0.38 | >0.05 |
| Sleep quality | **0.59** | **0.04** | **0.55** | **0.05** | 0.31 | >0.05 | **0.78** | **0.01** | **0.82** | **0.01** | 1 | - | 0.30 | >0.05 | **0.60** | **0.03** | 0.22 | >0.05 | 0.21 | >0.05 |
| Moderate levels of physical activity | -0.66 | 0.04 | -0.59 | 0.04 | 0.16 | >0.05 | 0.30 | >0.05 | 0.34 | >0.05 | 0.30 | >0.05 | 1 | - | -0.59 | 0.05 | 0.41 | >0.05 | 0.42 | >0.05 |
| Moderate levels of perceived stress | **0.59** | **0.03** | 0.33 | >0.05 | 0.25 | >0.05 | **0.56** | **0.03** | **0.57** | **0.04** | **0.60** | **0.03** | -0.59 | 0.05 | 1 | - | **0.52** | **0.05** | 0.23 | >0.05 |
| Intake of sugary foods | **0.76** | **0.01** | 0.21 | >0.05 | 0.27 | >0.05 | 0.36 | >0.05 | 0.33 | >0.05 | 0.22 | >0.05 | 0.41 | >0.05 | **0.52** | **0.05** | 1 | - | 0.40 | >0.05 |
| Consumption of fast meals | **0.67** | **0.01** | **0.50** | **0.04** | **0.59** | **0.04** | 0.35 | >0.05 | 0.38 | >0.05 | 0.21 | >0.05 | 0.42 | >0.05 | 0.23 | >0.05 | 0.40 | >0.05 | 1 | - |
| *Pearson coefficient correlation and* *Chi square test analysis. The bold indicates the positive significant correlations whereas the red indicates the negative correlations. Statistical significance attributed to results with p < 0.05.* | | | | | | | | | | | | | | | | | | | | |
